# P069 When weight isn’t the whole story: large vessel vasculitis with spondyloarthritis and a polymyalgic phenotype mistaken for fibromyalgia

**DOI:** 10.1093/rap/rkag113.099

**Published:** 2026-11-03

**Authors:** Thomas Hill, Benjamin Ellis

**Affiliations:** Imperial College Healthcare NHS Trust, London, United Kingdom; Imperial College Healthcare NHS Trust, London, United Kingdom

## Abstract

**Introduction:**

A 62-year-old male was reviewed in a Rheumatology clinic with a three-year history of widespread migratory pain and fatigue, previously diagnosed as fibromyalgia. Throughout this period, he had persistently mildly raised inflammatory markers, attributed to excess weight. Initial assessment raised concern for an underlying rheumatological cause, and investigation led to a diagnosis of large vessel vasculitis with axial spondyloarthritis and peripheral arthritis phenotype. This case highlights the pitfalls of attributing a persistent inflammatory response to obesity alone in people where fibromyalgia is suspected, and an important learning point regarding response to low-dose oral steroids not reliably excluding underlying autoimmune disease.

**Case description:**

A 62-year-old male was referred to a Rheumatology clinic with a three-year history of widespread migratory pains and fatigue, previously diagnosed as fibromyalgia, for which he had been under chronic pain services.

His background included type 2 diabetes, excess weight, post-traumatic stress disorder, depression, anxiety, left trigger finger (treated with multiple steroid injections), a previous left ankle fracture, sensorineural hearing loss, and current smoking.

Investigations via his GP showed persistently mildly raised inflammatory markers (CRP 20–25 mg/L, ESR 39–55 mm/hr) since 2023, with normal inflammatory markers prior to this. Anti-CCP, rheumatoid factor, ANA and ENAs were negative in 2024. Shoulder X-rays performed in 2024 were unremarkable. In 2024, a trial of oral steroid tablets (prednisolone 15mg) given for suspected polymyalgia rheumatica (PMR), gave no clear benefit.

On initial Rheumatology assessment, he had features consistent with right glenohumeral disease and supraspinatus tendinopathy, bilateral trochanteric bursitis, and tender swelling of both sternoclavicular joints, alongside right thumbnail dystrophy and mild scaling of the elbows and knees. There was no neurological deficit or cervical lymphadenopathy. He had marked difficulty rising from a chair.

MRI spine, sacroiliac joints and shoulders, and ultrasound of the shoulders and sternoclavicular joints, demonstrated sternoclavicular synovitis, unilateral sacroiliitis, mild bilateral subacromial bursitis, a right supraspinatus tear with bilateral supraspinatus tendinosis, and right glenoid full-thickness chondrosis with subchondral cystic change.

FDG PET-CT subsequently showed periarticular uptake in the shoulders, pelvic girdles, sternoclavicular joints, interspinous regions and sacroiliac joints, consistent with both polymyalgia rheumatica and mild sacroiliitis. Additionally, there was uptake in the thoracoabdominal aorta with a 73mm ascending aortic aneurysm.

He was subsequently diagnosed with spondyloarthritis with large vessel vasculitis with axial spondyloarthritis and peripheral arthritis phenotype. His symptoms dramatically improved with oral corticosteroids. Treatment was initiated with methotrexate and infliximab, followed by urgent surgical aneurism repair.

**Discussion:**

This case illustrates several important diagnostic pitfalls. Firstly, persistently mildly raised inflammatory markers may be attributed to obesity, given the low-grade inflammatory state associated with adipose tissue. However, obesity-related elevation in CRP/ESR should not be assumed as the sole explanation without investigation, particularly in symptomatic patients. In this case, the rise in CRP corresponded to the onset of symptoms, which should have prompted earlier consideration of an autoimmune or inflammatory disease rather than fibromyalgia.

Secondly, this case highlights the potential for overlap between large vessel vasculitis and a seronegative axial/peripheral inflammatory arthritis. The association between PMR and giant cell arteritis (a large vessel vasculitis) is widely familiar, and indeed this was a polymyalgic presentation with PMR features on PET-CT imaging. Large vessel vasculitis is an under-recognised association with a seronegative axial/peripheral inflammatory arthritis presentation and can be clinically silent. In this case, it was the findings of unilateral sacroiliitis, sternoclavicular arthritis and constitutional symptoms that led to the request for CT-PET.

Thirdly, the lack of response to a trial of standard-dose oral corticosteroids for suspected PMR had previously provided false reassurance against an inflammatory cause. This case demonstrates that partial or absent response to steroids should not be used in isolation to exclude an underlying autoimmune or inflammatory process, particularly when dosing, duration, or the underlying pathology (e.g. large-vessel vasculitis, axial spondyloarthritis) may not respond well to standard PMR-dose corticosteroids.

Finally, this case underscores the value of FDG PET-CT in patients with atypical or overlapping symptoms, as it identified occult large-vessel vasculitis that would not otherwise have been detected, requiring urgent medical and surgical management.

**Key learning points:**

• Although fibromyalgia is not a diagnosis of exclusion, abnormal features in the history, clinical examination or screening investigations should always prompt a consideration for other causes of symptoms; like the general population, people with fibromyalgia are at risk of developing systemic autoimmune/inflammatory disease, and a change or worsening in symptoms should lead to careful review for new or comorbid conditions.

• Avoid attributing persistently elevated inflammatory markers to excess weight alone. Obesity can cause raised CRP/ESR, but should be the assumed cause, particularly in the symptomatic patient when the timing of CRP rise correlates with the onset of new symptoms. Ongoing or worsening elevation should prompt re-evaluation for an underlying inflammatory or autoimmune cause.

• Poor or partial response trial of PMR-dose oral corticosteroids does not rule out an autoimmune or inflammatory diagnosis. A negative response to steroids for suspected PMR delayed this patient’s diagnosis; corticosteroid response can be affected by metabolic factors, corticosteroid dose and duration, and the specific underlying pathology.

• Polymyalgic symptoms can be a presenting phenotype of spondyloarthritis, particularly in older adults and when accompanied by peripheral synovitis including sternoclavicular joints, axial disease including sacroiliitis, and skin/nail changes suggestive of a psoriasiform process.

• Large vessel vasculitis including aortitis is an important potentially life-threatening association with both PMR and spondyloarthritis, and may be clinically silent. Cardiovascular imaging should be considered in patients with an atypical inflammatory presentation, particularly with overlapping axial and polymyalgic features, and systemic constitutional features

• FDG PET-CT can help in patients with diagnostic uncertainty and overlapping rheumatological phenotypes, identifying typical PMR features and detecting occult large-vessel vasculitis that may otherwise go unrecognised.

